# Supplementary material for: Preeclampsia, prevalence and associated factors
Source: PLoS One. 2025 Dec 4;20(12):e0337190. doi: 10.1371/journal.pone.0337190 (PMC12677571; doi:10.1371/journal.pone.0337190)
Supplement: Attachment — Submitted filename: Responses to Reviewers.pdf [file pone.0337190.s005.pdf]

29/09/2025

PLOS ONE Journal

Dear Editor,

Ref: Submission of a revised research article for peer review and publication consideration

Reference to the above-mentioned subject. I am writing to submit a revised original research article titled "**Preeclampsia, prevalence and associated factors**,". We would like to thank the reviewers for taking the time to make suggestions that have improved our manuscript. We have revised the manuscript and addressed all concerns and suggestions. We now hope the current manuscript is acceptable for publication. Below are the point-by-point responses to all comments and suggestions

Response to the reviewers

Background Review - Scientific Rigor and Relevance
Reviewer: Specificity of sources: While references are cited, clarity in source attribution is important. More precise statements referencing current and key studies would enhance credibility.
Response: We thank the reviewer for this critical observation. We have thoroughly revised the background section to incorporate more specific and recent references. Key statements on the global and regional burden of PE have been updated with precise citations from recent studies (within the last 5 years where available) to strengthen the credibility and context of our work [Updated Refs 2-6]. We have also ensured that citations for the clinical definition [Ref 1] and pathophysiological mechanisms [Refs 7-9] are clear and authoritative.

Reviewer: Balance: The section leans heavily on theoretical associations but lacks sufficient grounding in empirical population data for LMICs.
Response: We agree. We have revised the background to better contextualize known risk factors within the LMIC and Zambian setting. We have added a sentence to explicitly state the scarcity of comprehensive data from regions like Southern Zambia and have strengthened the rationale for our study by highlighting that it aims to provide this missing empirical, population-based evidence from a high-burden setting to inform local clinical practice and policy.

Reviewer: Language and Grammar corrections (multiple specific examples provided).
Response: We have incorporated all these language and grammar corrections into the revised manuscript to improve clarity, precision, and academic tone.

Methods Section
Reviewer: Grammar and syntax corrections are needed.
Response: Thank you. We have revised the grammar and syntax throughout the methods section.

Reviewer: Sample size calculation requires more clarity on assumptions.
Response: Thank you. We have clarified the sample size calculation by explicitly stating the

parameters used: an expected frequency of 7.7%, a 95% confidence level (Z=1.96), a margin of error of 5%, and a design effect of 1. The formula has been included in the text for transparency.

Reviewer: Systematic sampling method needs clarification.
Response: Thank you. We have clarified the sampling strategy. The list of patient records was ordered by date of visit. The starting point was then randomly selected before selecting every third file, which is a standard systematic sampling approach.

Reviewer: Grouping of variables will improve readability.
Response: Thank you. We have now grouped the independent variables into clear categories: Sociodemographic, Obstetric and reproductive history, Clinical, and Laboratory haematological variables.

Reviewer: Data collection period should be checked for temporal consistency.
Response: We sincerely apologize for this critical error. The correct data collection period was 1st December 2023 to 30th March 2024. This has been corrected in the manuscript.

Results Section
Reviewer: Language and Grammar corrections (multiple specific examples provided).
Response: Thank you. We have revised all the indicated grammar, punctuation, and formatting issues for clarity and professionalism (e.g., "from different health facilities," "Univariable," "21.4%," consistent spacing and units).

Reviewer: The results would benefit from more narrative synthesis.
Response: We have added a stronger narrative summary at the beginning of the results section to better highlight and interpret the key findings in context.

Reviewer: Multivariable Model Reporting: Please state how variables were selected for inclusion.
Response: We have added the following explanation to the methods section: "Variables with a p-value < 0.01 in the univariable analysis, along with those of known clinical importance, were considered for inclusion in the multivariable logistic regression model. We also excluded variables with significant collinearity. A backward stepwise selection process was used to arrive at the final adjusted model."

Reviewer: Statistical Accuracy: Contradictory statement regarding gestational age odds ratio.
Response: Thank you for identifying this error. We have corrected the narrative to accurately reflect that an increase in gestational age is associated with *decreased* odds of PE (OR < 1).

Discussion and Conclusion
Reviewer: While findings are well-discussed, it could benefit from a more analytical tone evaluating the strength of associations and clinical relevance.
Response: We have revised the discussion to more critically analyze the strength of our high odds ratios (e.g., AOR of 30.8 for prior history) and their profound clinical relevance. We have

added a specific recommendation on integrating family and obstetric history into antenatal risk stratification.

Reviewer: Language and Writing Quality: Minor grammatical errors and repetitive phrasing.
Response: Thank you. We have corrected the grammatical errors (e.g., "unexplained and idiopathic"), eliminated repetitive phrasing, broken up long paragraphs, and improved overall clarity.

Reviewer: Limited commentary on public health or clinical implications.
Response: We have added a sentence in the discussion to address this: "Incorporating routine assessment of prior and family history of PE into antenatal risk stratification may facilitate early detection and reduce adverse maternal outcomes."

Reviewer: Conclusion: Make it slightly more actionable.
Response: We have revised the conclusion as suggested: "These findings support the integration of targeted screening tools into maternal health programs and underscore the need for more prospective cohort studies to explore causality and intervention strategies."

Reference Section
Reviewer: Remove duplicate references and correct journal name formatting.
Response: Thank you. We have removed the duplicate entries for Karrar et al. and Mukosha et al. We have also reformatted all journal titles to their full names (not abbreviated) and ensured consistency with the Vancouver/PLOS ONE style throughout the reference list.
