# Supplementary material for: Preeclampsia, prevalence and associated factors
Source: PLoS One. 2025 Dec 4;20(12):e0337190. doi: 10.1371/journal.pone.0337190 (PMC12677571; doi:10.1371/journal.pone.0337190)
Supplement: Attachment — Submitted filename: reviewer comments.docx [file pone.0337190.s003.docx]

# **Background review**

**Scientific Rigor and Relevance**

*Strengths:*

The section clearly introduces preeclampsia (PE) as a global maternal health concern and outlines its clinical definition and consequences.

The text identifies both clinical and molecular risk factors, emphasizing the multifactorial nature of PE.

The relevance of genetic, immunological, environmental, and lifestyle factors is well-articulated and aligns with current literature.

*Areas for Improvement:*

Specificity of sources: While references are cited (e.g., [1], [2]), in PLOS ONE, clarity in source attribution is important. More precise statements referencing current and key studies (preferably within the last 5 years) would enhance credibility.

Balance: The section leans heavily on theoretical associations (e.g., genetics, immunology) but lacks sufficient grounding in empirical population data, particularly for low- and middle-income countries (LMICs), despite acknowledging their burden.

**Language and Grammar**

Corrections/Suggestions:

Line 1: “fatal health” should be “fetal health”

Line 2: “may lead to rapid development of severe complications” consider “can rapidly progress to severe complications”

Line 4: “where access to specialized care often falls short” revise to “where access to specialized obstetric care is often limited”

Line 6: “external contributors like lifestyle choices” ,replace with “modifiable factors such as lifestyle behaviors”

Line 9: “emphasize the hereditary and epigenetic dimensions” consider “highlighting hereditary and epigenetic contributions”

Line 13: “dissecting these complex interactions” ,could be simplified to “understanding these interactions”

**Overall Recommendation for Background**

The background is well-conceived, informative, and suitable for a PLOS ONE audience with minor revisions needed for grammar, conciseness, and citation clarity. Incorporating a clear research gap statement especially tied to regional context (e.g., Sub-Saharan Africa or your study’s setting) will help justify the importance of the study more explicitly.

## **Methods Section**

### ***Strengths***

- Follows appropriate structure: study design, setting, eligibility, variables, data collection, analysis, and ethics.
- Provides clear definitions for key variables such as pre-eclampsia and HELLP syndrome.
- Describes sampling strategy, variable selection, and statistical approach.
- Includes ethical considerations in line with standard research practices.

### **Areas for Improvement (Aligned with PLOS ONE Guidelines)**

| **Section** | **Issue** | **Recommendation** |
| --- | --- | --- |
| **Grammar** | Several typos (e.g., "form" instead of "from", "patient’s" instead of "patients'") and punctuation issues | Proofread carefully and apply consistent academic language. |
| **Sampling strategy** | Method described as "systematic sampling," but selection from medical records every third file may be biased | Clarify whether files were sorted or randomized to reduce systematic error. |
| **Sample size** | Mentions OpenEpi and 7.7% prevalence, but lacks formula or assumptions used | Include the sample size calculation formula or refer explicitly to the parameters used. |
| **Variable listing** | Long list presented without organization | Group variables into categories (sociodemographic, clinical, laboratory, etc.) for readability. |
| **Data collection dates** | “1st December 2024 to 30th March 2025” – ensure temporal consistency and logic in context (if manuscript is submitted before or after these dates) | Double-check if this range is realistic relative to current time. |
| **Clarity of definitions** | Some definitions lack citation formatting or are run-on | Use clear definitions with numbered citations and paragraph breaks. |

## **Overall Recommendation: Minor Revision**

The Methods section is **scientifically sound,** adequately structured, and includes all required components (study design, population, variables, data collection, analysis, and ethics). The section demonstrates a solid foundation for a rigorous and reproducible study. However, **minor revisions** are required for clarity, consistency, and adherence to formatting and reporting standards expected in PLOS ONE.

### ***Strengths***

- Clearly stated the **study design**, **setting**, and **eligibility criteria**.
- Included **definitions** for clinical outcomes (PE and HELLP syndrome).
- Provided **ethical approval details**, which align with international standards.
- Used appropriate **statistical techniques** (normality tests, regression models).
- Utilizes **REDCap** and validated data collection tools.

***Minor Issues to Address***

1. **Grammar and syntax corrections** are needed throughout to improve flow and professionalism.
2. **Sample size calculation** requires more clarity on assumptions used (confidence level, power, design effect).
3. **Systematic sampling** method needs clarification—especially whether files were ordered or randomized.
4. **Grouping of variables** will improve readability and reduce repetition.
5. **Data collection period** should be checked for temporal consistency (dates should make sense in relation to the manuscript’s submission timeline).

**Review of Results**

Strengths

Clear participant flow: The inclusion of a total starting sample and reasons for exclusion (2,582 excluded from 3,600) is well-presented. This aligns with PLOS ONE's expectation for transparency in participant selection.

Descriptive statistics are thorough and cover a wide range of variables, including sociodemographic, clinical, and laboratory data.

Univariable and multivariable analyses are reported with odds ratios, confidence intervals, and p-values in line with best practices.

The presentation of Figure 1 and Figure 2 is appropriate and adds clarity .

Tables are data-rich and well-organized, allowing readers to draw meaningful comparisons between participants with and without PE.

***Areas need Improvement***

*Language and Grammar*

There are several grammar, punctuation, and formatting issues that affect clarity. For example:

“form different health facilities” should be “from different health facilities”

“Unavailable and multivariable analysis” should be “Univariable and multivariable analysis”

"21.4" in “21 (21,4)” likely a formatting error; use “21 (21.4%)”

Typographic inconsistencies like “ALT;Alanine transferase” and spacing errors (e.g., “9.97 95%Cl”) should be corrected for clarity and professionalism.

**Data Presentation**

The results would benefit from more narrative synthesis. Many statistics are listed, but key findings are not sufficiently highlighted or interpreted in context.

Some figures such as "Haemoglobin, gram/dl" show inconsistent values (e.g., “31 (25, 34)” seems to be an error).

Variable names like "Gestational_age_weeks_2" need to be renamed for clarity in the manuscript.

Multivariable Model Reporting

Only some variables from the univariable analysis were included in the multivariable model but it is unclear how selection was done (e.g., p-value < 0.2 threshold or stepwise regression?). For transparency, please state how variables were selected for inclusion in the adjusted model.

**Statistical Accuracy and Interpretation**

Some statements appear mathematically unclear or misleading:

“An increase in gestational age increased the odds of having PE by 11% (OR: 0.86...)” This is contradictory. OR <1 indicates decreased odds, not increased.

Double-check use of terms like "increase" vs. "decrease" with the directionality of ORs.

**Figure 2 Descriptions**

Figure 2 is described in extensive detail, but the interpretation is not included. Consider summarizing what was observed e.g., "Women with PE had significantly lower gestational age and higher blood pressure, while hematological parameters did not significantly differ."

***Recommendations for Revision***

Use uniform formatting for:

p-values (e.g., "p < 0.001", not "P=0.0001" or “p<0.0.1”)

Units (e.g., "g/dL", "µmol/L", "x10³/µL")

Ensure tables are consistently formatted, especially with missing or mismatched values.

Clarify variable groupings (sociodemographic, clinical, lab).

Rename ambiguous variable labels (e.g., "Gestational_age_weeks_2").

## **Review of Discussion and Conclusion**

**Strengths:**

- **Well-articulated interpretation** of major findings (e.g., association of PE with age, gestational age, and family history).
- **Strong linkage to previous literature**, including global and regional comparisons, especially in the African context.
- Provides **biological plausibility** and **mechanistic discussion** (e.g., placental dysfunction, epigenetics, immune maladaptation).
- Inclusion of a **robust limitation section** is commendable and expected in PLOS ONE.

**Areas for Improvement:**

- While findings are well-discussed, the **discussion is descriptive**. It could benefit from a more **analytical tone** by evaluating:
  - The **strength** of associations (e.g., AORs of 9.97 and 30.8).
  - The **clinical relevance** of variables (e.g., should family history be included in all antenatal intake forms?).
- The **pathophysiological explanation** of PE recurrence is strong, but some points (e.g., “deoxyribose nucleic acid” instead of simply “DNA”) can be simplified for readability.

### ***Language and Writing Quality***

**Strengths:**

- The flow is logical and follows a standard scientific structure: findings ,comparison with literature , explanation ,limitations ,conclusion.
- Technical terminology is used appropriately.

**Weaknesses:**

- Minor grammatical and stylistic errors:
  - e.g., “unexplained and idiopathic explaining why.” awkward phrasing.
  - e.g., “another variable that emerged…” repetitive phrasing.

**Suggestions:**

- Use clearer phrasing: “Our study revealed a slightly higher prevalence (12.7%) than Mukosha et al. (12%), although both findings are largely consistent.”
- Avoid redundancy: “history of PE was significantly associated with recurrence” and “prior PE are at increased risk” can be merged.
- Break long paragraphs into smaller ones to improve readability, especially the third paragraph.

***Context and Relevance***

**Strengths:**

- Excellent integration of findings within regional and global literature.
- Clear identification of gaps in local epidemiological data (Zambia and SSA focus).
- Use of citations is extensive and well-referenced.

**Weaknesses:**

- Limited commentary on **public health or clinical implications**.
  - For instance: how can the findings inform **antenatal screening, referral policies, or provider training**?

**Suggestions:**

- Add a line such as:“Incorporating routine assessment of prior and family history of PE into antenatal risk stratification may facilitate early detection and reduce adverse maternal outcomes.”

***Limitations and Strengths Section***

**Strengths:**

- Balanced and honest assessment of the study’s limitations.
- Clearly acknowledges retrospective design, data completeness, testing methods, and generalizability.
- Highlights methodological strengths such as large sample size, data verification, and variable inclusion.

**Suggestions:**

- Move the sentence on sample size and data verification closer to the beginning of the paragraph to maintain balance.

***Conclusion Section***

**Strengths:**

- Clearly summarizes the main predictors of PE.
- Emphasizes the need for future research into mechanisms and interventions.

**Suggestions:**

- Make the conclusion slightly more actionable. Consider:

“These findings support the integration of targeted screening tools into maternal health programs and underscore the need for more prospective cohort studies to explore causality and intervention strategies.”

## ***Summary of Recommendations***

### Revise the following:

- Improve clarity in phrasing and grammar throughout the discussion.
- Reduce repetition (e.g., repeated "another variable that emerged").
- Strengthen the **interpretation of effect sizes** and their clinical relevance.
- Add one or two sentences about **implications for practice or policy** in the conclusion.

Reference section

Your reference section is comprehensive and well-sourced. However, for submission to PLOS ONE, it must follow the journal’s strict formatting guidelines, which are based on Vancouver style, with specific expectations. Below is a detailed review and recommendations to align your reference section with PLOS ONE formatting standards:

What You Did Well

You included DOIs or URLs for almost all sources, which is strongly encouraged by PLOS ONE.

The references are rich in recent studies (2020–2024), indicating your literature review is up to date.

Key topics (e.g., pathophysiology, prevalence, genetic factors) are well-referenced with peer-reviewed journal articles.

Issues to Correct

1. Remove Duplicate References

Example:

Karrar SA, Martingano DJ, Hong PL. Preeclampsia. StatPearls... appears twice.

→ Remove one instance and renumber.

Same for:

Mukosha M, Vwalika B, Lubeya MK, Kumwenda A, Kaonga P, Jacobs C, et al.

Appears twice, also needs deduplication.

2. Correct Journal Names and Formatting

Use full journal titles in title case, not abbreviations or partial capitalizations.

Correct: American Journal of Obstetrics and Gynecology

Incorrect: Am J Obstet Gynecol

PLOS ONE style requires no italics or bold, no quotation marks, and no brackets around volume or page numbers.

Ensure Consistent Author Formatting

List up to 6 authors, then use et al. after the 6th.

Correct: Smith A, Jones B, Chen C, Patel D, Kumar R, Tan L, et al.

Incorrect: Smith A, Jones B, Chen C, et al., Patel D

Use Proper Structure for Each Entry

Each reference should follow this exact order:

Author(s). Article title. Journal Title. Year;Volume(Issue):Pages. doi:DOI

Example (Correctly formatted):

Rana S, Lemoine E, Granger JP, Karumanchi SA. Preeclampsia: Pathophysiology, challenges, andperspectives.CirculationResearch.2019;124(7):10941112.doi:10.1161/CIRCRESAHA.118.313276
